# Regulation of lipid oxidation and ferroptosis by a tunable axis of vitamin E, high-density lipoprotein, and scavenger receptor class B type 1

**DOI:** 10.1016/j.jbc.2026.113497

**Published:** 2026-08-27

**Authors:** Jonathan S. Rink, Andrea E. Calvert, Sophia M. Lamperis, Alexandra Moxley, Adam Yuh Lin, SonBinh T. Nguyen, Leo I. Gordon, C. Shad Thaxton

**Affiliations:** 1 Department of Urology, Northwestern University Feinberg School of Medicine, Chicago, Illinois, USA; 2 Division of Hematology/ Oncology, Department of Medicine, Northwestern University Feinberg School of Medicine, Chicago, Illinois, USA; 3 Robert H. Lurie Comprehensive Cancer Center, Northwestern University, Chicago, Illinois, USA; 4 Department of Chemistry, Northwestern University, Evanston, Illinois, USA; 5 Jesse Brown Veteran Affairs Medical Center, Chicago, Illinois, USA

**Keywords:** ferroptosis, α-tocopherol, high-density lipoproteins, SR-B1, oxidation

## Abstract

Ferroptosis is caused by lethal peroxidative damage to cell membrane phospholipids (PL) containing polyunsaturated fatty acid (PUFA) tail groups. Mechanisms to control cell membrane PL oxidation and ferroptosis include cellular expression of glutathione peroxidase 4 (GPx4), which repairs oxidized cell membrane PL, and the lipophilic antioxidant alpha-tocopherol (α-toc), an active form of Vitamin E. Data show that α-toc can rescue cells from ferroptosis *in vitro* and *in vivo* even in the absence of GPx4. Yet, pathways by which α-toc is transported in blood, for instance in high- and low-density lipoproteins (HDL and LDL), for delivery to target cells to prevent PL oxidation and ferroptosis are unknown. We hypothesized that HDLs specifically target delivery of α-toc to the cell membrane to regulate PL oxidation and sensitivity to ferroptosis. We employed native lipoproteins and synthetic HDL-like nanoparticles made using either organic or gold nanoparticle cores (ocHDL NP or Au-HDL NP, respectively). The bottom-up synthesis strategies enabled control over the physicochemical properties of HDL NP, including α-toc content. Using cancer and neuronal cell models, our data show native HDLs and synthetic HDL NPs deliver α-toc by binding cell membrane receptor scavenger receptor class B type 1 (SR-B1) to prevent PL oxidation and ferroptosis. Additionally, data show Au-HDL NP, previously shown to bind SR-B1, reduce GPx4, and induce ferroptosis can rescue cells from ferroptosis when synthesized to contain α-toc. Overall, our data demonstrate a tunable cellular redox axis whereby HDLs containing α-toc target SR-B1 to regulate sensitivity to ferroptosis.

Ferroptosis is an iron-dependent cell death mechanism that results from shifting cellular redox balance to favor increased lipid reactive oxygen species (L-ROS) and the propagation of peroxidative (-OOH) damage to polyunsaturated fatty acid (PUFA) tail groups of cell membrane phospholipids (PLs) (1, 2, 3). There is considerable interest in manipulating cellular PL redox balance and ferroptosis for next generation cancer therapies and to prevent neurodegeneration (4, 5). Thus, better understanding relevant mechanisms of controlling PL oxidation and ferroptosis is of considerable importance.

Efforts to define mechanisms of ferroptosis have leveraged genetic screening techniques to identify cell endogenous regulatory proteins and pathways [reviewed in (6)]. The active form of Vitamin E, alpha-tocopherol (α-toc), is a cell exogenous lipophilic radical trapping antioxidant (RTA) that localizes to the cell membrane to reduce lipid oxidative damage (7, 8, 9). *In vitro* studies focused on pathways to induce ferroptosis in cancer cells by targeting the cystine/glutathione (GSH)/GPx4 axis established that α-toc rescues cells from cell death even when protective pathways have been inhibited (10, 11, 12). Importantly, in *in vivo* animal models, α-toc has been shown to prevent oxidative cell death in multiple cell types, including neurons (8, 13, 14, 15) and hematopoietic stem cells (16), when the lipid protective functions of GPx4 have been reduced or genetically knocked out (13, 14, 15, 16). While the protective effects of α-toc against ferroptosis are well known, precise mechanisms by which α-toc is delivered to cells to prevent ferroptosis has yet to be described.

As a lipophilic antioxidant, α-toc requires solubilization in high- and low-density lipoproteins (HDL and LDL) to be transported in the blood stream (17, 18). Mature HDLs bind to their high affinity cell surface receptor scavenger receptor class B type 1 [SR-B1, (19)] while LDLs predominantly target the LDL receptor [LDLR; (20)]. It is unknown whether α-toc carried by lipoproteins target cells and regulate sensitivity to ferroptosis. To investigate α-toc, lipoproteins, and lipoprotein-relevant cell surface receptors as mediators of ferroptosis, we employed native HDL and LDL, including lipoproteins manipulated to have higher α-toc content. Additionally, we developed synthetic, organic-core templated HDL-like nanoparticles (ocHDL NP) with similar size, shape (spherical), charge (anionic) and surface chemistry [*e.g.*, apolipoprotein A-I (apoA-I) and phospholipids] as native HDLs that bind to SR-B1 (21). The ocHDL NPs enabled us to control particle composition, including α-toc content. Ultimately, native and synthetic lipoproteins deliver α-toc to target cells by binding SR-B1. Delivered α-toc inhibits cell membrane lipid oxidation and ferroptosis in cancer cells and in neuronal cells. Further, delivery of α-toc using HDL NP synthesized using an inert gold nanoparticle core (*i.e.*, Au-HDL NP) rescued cells from ferroptosis despite also functioning to significantly reduce GPx4. These data delineate an α-toc/HDL/SR-B1 axis of cellular protection against cell membrane lipid peroxidation that can be purposefully manipulated using native and synthetic ligands of SR-B1 to regulate ferroptosis.

## Results

### Native and synthetic SR-B1 ligands rich in α-toc rescue cancer cells from ferroptosis

For these studies, we employed a definition of ferroptosis that included cell death, increased lipid peroxidation, and rescue by established ferroptosis inhibitors [*e.g.*, lipophilic antioxidants and iron chelators; (6)]. To induce ferroptosis, we employed two molecules, erastin and ML210, which are well-established to induce ferroptosis (22, 23, 24). Diffuse large B-cell lymphoma (DLBCL) and clear cell renal cell carcinoma (ccRCC) cell lines were among the first shown to be particularly sensitive to ferroptosis induced by inhibiting system x_c_^−^ (erastin) or GPx4 [ML210; (9)]. Consistent with these findings, our group demonstrated that the B-cell lymphoma cell lines SUDHL4 (DLBCL) and Ramos (Burkitt’s lymphoma) are sensitive to ferroptosis (25), and prior studies have shown that the ccRCC cell line, 786-O, is sensitive to ferroptosis (5). Thus, we explored α-toc delivery by lipoproteins and ferroptosis in cancer cells using Ramos, SUDHL4, and 786-O cells. Data confirmed that these cell lines express SR-B1 (Fig. S1*A*) and the LDLR (Fig. S1*B*). Experiments were conducted in standard cell culture media containing serum [*i.e.*, fetal bovine serum (FBS)] rather than lipoprotein-depleted serum (LPDS) to better represent physiologic conditions.

We investigated whether native HDL or LDL rescues the cancer cell lines from cell death induced by ML210 (24). First, we measured the amount of α-toc in native HDL and LDL particles per apolipoprotein [apolipoprotein A-I (apoA-I) for HDL and apolipoprotein B-100 (apoB-100) for LDL]. Native HDL particles had ∼1.0 ± 0.1 α-toc molecule per apoA-I, and LDL particles had 6.0 ± 0.03 α-toc molecules per apoB-100 (Table S1), consistent with previous reports (26, 27, 28). To increase the α-toc content of native lipoproteins, we incubated HDL and LDL with free α-toc followed by particle purification. The resultant α-toc lipoproteins (α-toc HDL and α-toc LDL) showed an increase in α-toc to apolipoprotein ratios (1.6 ± 0.3 per apoA-I in α-toc HDL and 10.1 ± 0.1 per apoB-100 in α-toc LDL; Table S1).

We treated the Ramos, SUDHL4, and 786-O cancer cells with a dose of ML210 that induced >95% cell death in each cell line (250 nM for Ramos; 100 nM for SUDHL4; 1 μM for 786-O; Fig. S2, *A–C*). Additionally, we added equimolar concentrations of native HDL and LDL, and α-toc HDL and α-toc LDL (Fig. 1, *A*–*C*), based on particle concentration. We found that HDL rescued the cancer cells from ML210-induced cell death at lower concentrations compared with LDL (Fig. 1, *A*–*C*) even though HDL contained significantly less α-toc per particle compared to LDL (Table S1). Both the α-toc HDL and α-toc LDL demonstrated an enhanced ability to rescue ML210-induced cell death (Fig. 1, *A*–*C*). We employed the SUDHL4 cells to confirm that rescue from cell death was accompanied by a decrease in cell membrane lipid peroxide accumulation after treatment with the various lipoproteins. As expected, increased cell viability was accompanied by reduced cell membrane lipid oxidation measured using C11-BODIPY (Fig. 1*D*).

**Figure 1:**
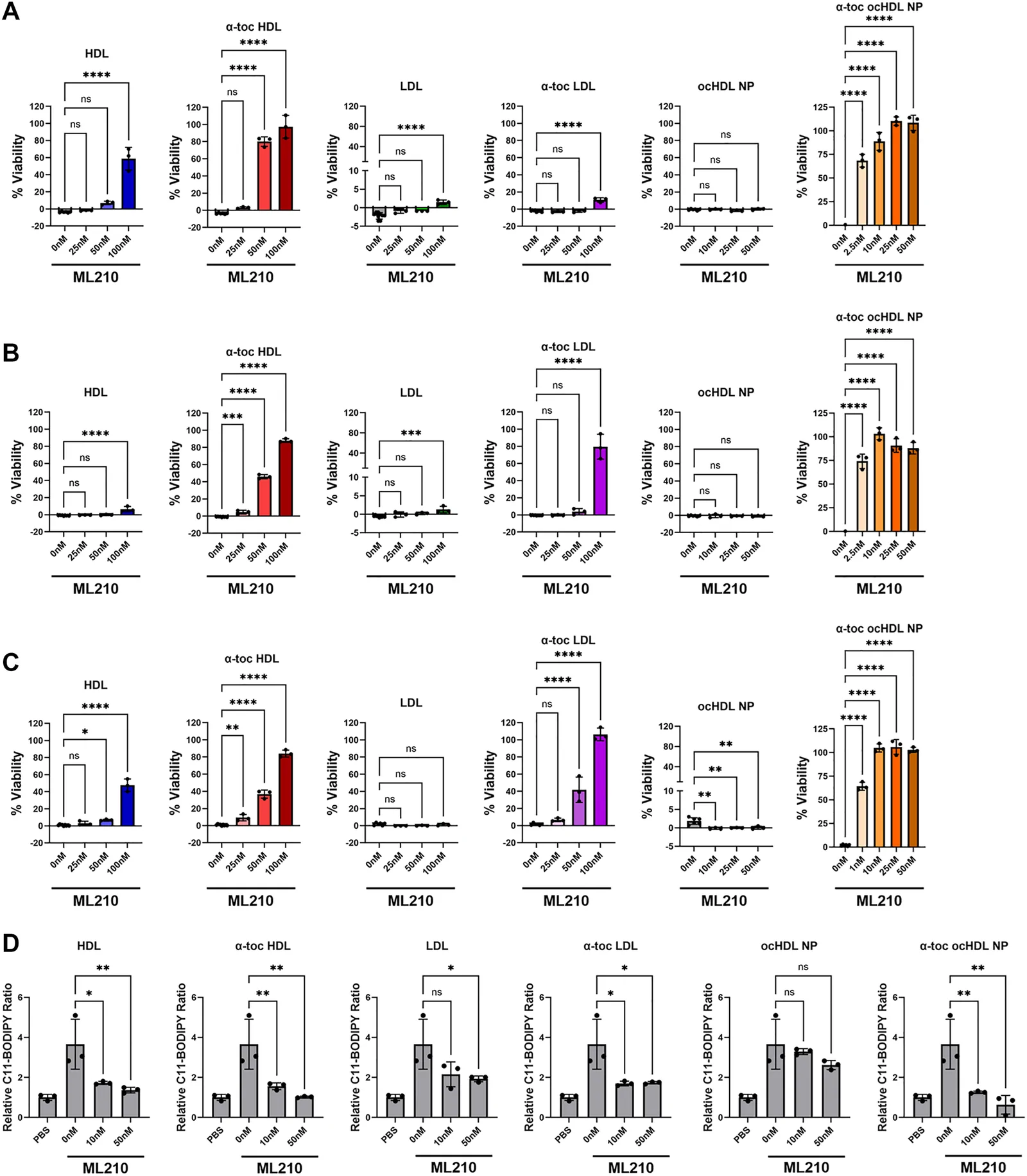
**α-toc delivery by natural lipoproteins rescues ML210-induced cell death in DLBCL and ccRCC cells.***A–C*, ramos (*A*), SUDHL4 (*B*), and 786-O (*C*) cells were treated with ML210 (Ramos – 250 nM, SUDHL4 – 100 nM; 786-O – 1 μM) and increasing concentrations of HDL, α-toc HDL, LDL, α-toc LDL, ocHDL NP and α-toc ocHDL NP for 72 h. *D*, C11 BODIPY analysis of lipid peroxide accumulation in SUDHL4 cells treated with ML210 (100 nM) and HDL, α-toc HDL, LDL, α-toc LDL, ocHDL NP and α-toc ocHDL NP. Statistics: 1-way ANOVA with Tukey *post hoc* analysis. ∗*p* < 0.05, ∗∗*p* < 0.01, ∗∗∗*p* < 0.001 and ∗∗∗∗*p* < 0.0001 indicate statistical significance. No statistical difference (*p* > 0.05) is noted by “ns.”

Native HDLs are dynamic nanostructures and chemically heterogeneous (29, 30, 31). Therefore, to isolate the effect of α-toc delivered by HDL, we utilized a synthetic HDL NP platform where particle composition could be controlled. This synthetic HDL NP platform utilizes an organic core (oc) molecule that serves as a core template to support the self-assembly of phospholipids and apoA-I into particles, ocHDL NP (21). The ocHDL NPs have compositional (*e.g.*, apoA-I and phospholipids) and physical (*e.g.*, size, shape, negative surface charge) similarities to native HDL and target SR-B1 (21). We synthesized ocHDL NP with and without α-toc. The resultant α-toc ocHDL NPs had 3.5 ± 2.2 α-toc per apoA-I, which exceeds α-toc levels in natural HDLs or α-toc HDLs. This is due to the increased core volume in each ocHDL NP available for α-toc loading *versus* in native HDLs where neutral lipid cargo (*e.g.*, cholesterol ester and triglycerides) occupies the core (Table S1). The ocHDL NP and α-toc ocHDL NPs trended toward being larger than native HDL and α-toc HDL, which may have also contributed to higher α-toc loading, but the sizes were not statistically significantly different (Table S1). The α-toc ocHDL NPs did not differ in size compared with empty ocHDL NPs (*p* = 0.9934; Table S1); however, the surface charge (zeta potential) of α-toc ocHDL NPs was significantly less negative than that of empty ocHDL NPs (Table S1). The less negative zeta potential in α-toc ocHDL NP may be due to packing of neutral α-toc in the ocHDL NP, alterations in the assembly of phospholipids, and/or apoA-I conformation upon α-toc loading (32, 33). We utilized the ocHDL NPs to investigate the delivery of α-toc and the effects of the materials on PL oxidation and ferroptosis.

As in the previous experiments, we added increasing concentrations of ocHDL NPs or α-toc ocHDL NPs to the DLBCL and ccRCC cell lines treated with ML210. We found that ocHDL NPs without α-toc did not rescue the cell lines from ML210-induced cell death; however, addition of the α-toc ocHDL NPs led to a significant increase in cell survival (Fig. 1, *A*–*C*). Cell rescue was accompanied by a significant decrease in cell membrane lipid oxidation measured using C11-BODIPY (Fig. 1*D*).

To confirm these findings using an alternative mechanism of cystine/GSH/GPx4 inhibition, we repeated these experiments using the ferroptosis inducer, erastin. Erastin inhibits cellular uptake of the GSH precursor, cystine, through system x_c_^−^ as a well-established inducer of ferroptosis (22). We found that both natural and synthetic lipoproteins loaded with α-toc rescued the cells from erastin-induced ferroptosis (Fig. 2).

**Figure 2:**
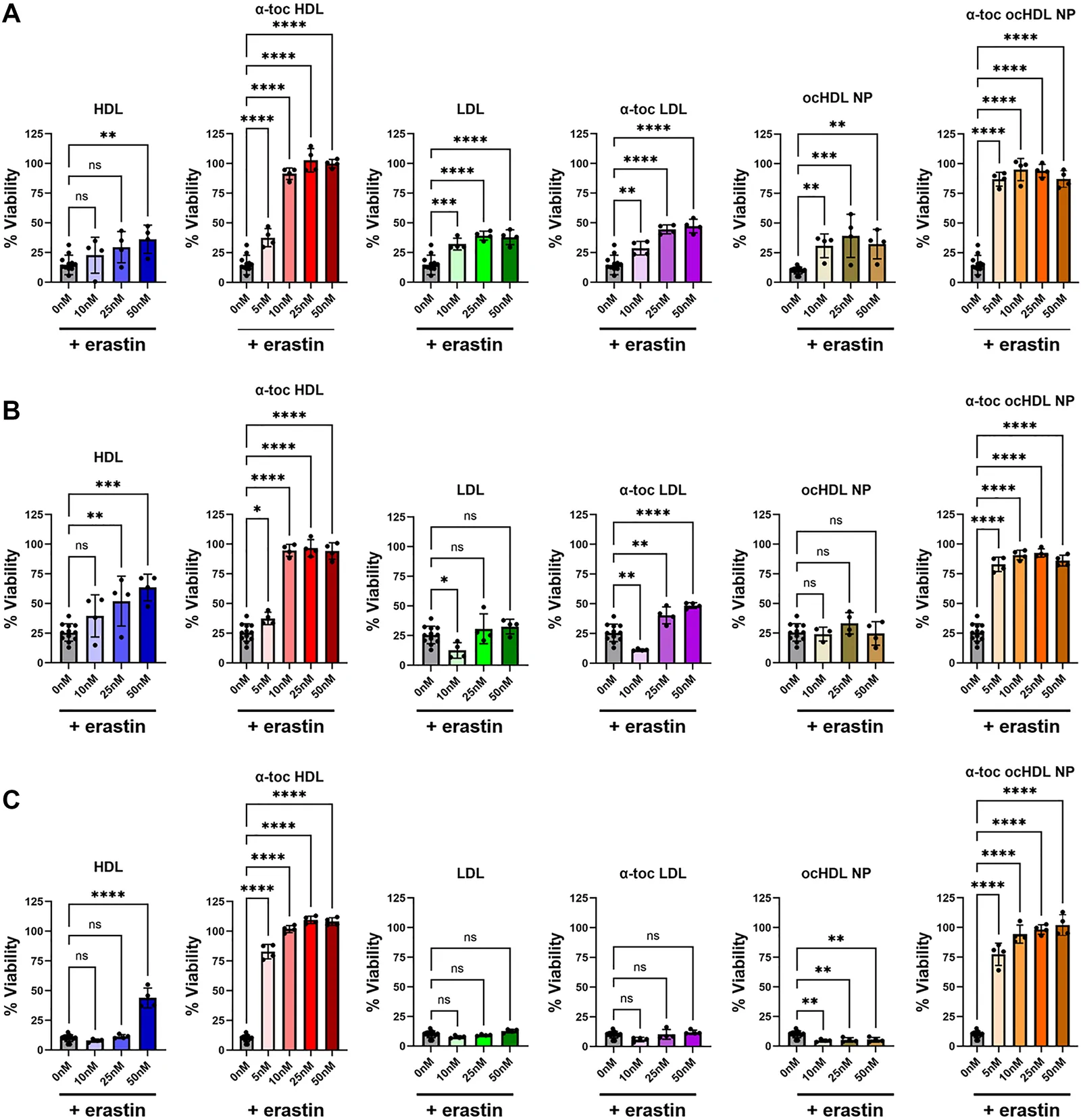
**Lipoprotein rescue of erastin-induced cell death.***A–C*, Rescue of Ramos (*A*), SUDHL4 (*B*), and 786-O (*C*) cells from erastin-induced cell death by HDL, α-toc HDL, LDL, α-toc LDL, ocHDL NPs, and α-toc ocHDL NPs. Statistics: 1-way ANOVA with Tukey *post hoc* analysis. ∗*p* < 0.05, ∗∗*p* < 0.01, ∗∗∗*p* < 0.001 and ∗∗∗∗*p* < 0.0001 indicate statistical significance. No statistical difference (*p* > 0.05) is noted by “ns.”

### SR-B1 mediates uptake of α-toc from lipoproteins

Both HDLs and LDLs can bind SR-B1 and the LDLR (19, 34). Furthermore, both receptors have been shown to be expressed in a number of different malignancies, including DLBCL and ccRCC (35, 36). To assess the role of the individual receptors in lipoprotein-mediated α-toc delivery and ferroptosis, we used siRNA to reduce expression of SR-B1 and LDLR, and we used the small-molecule inhibitor of molecular transport through SR-B1 called BLT-1 [blocker of lipid transport-1 (34)]. BLT-1 blocks SR-B1-mediated molecular transport of lipoprotein cargo into the cell, including α-toc (37), but does not inhibit lipoprotein binding to SR-B1 (38). For siRNA experiments, we used the adherent 786-O cells as they are more conducive to siRNA transfection than the DLBCL cell lines that grow in suspension. Ramos, SUDHL4 and 786-O cells were amenable to study using BLT-1.

To investigate delivery of α-toc by lipoproteins *via* binding and delivery through SR-B1, we first reduced SR-B1 expression in 786-O cells using siRNA. Consistent with our previous data in ovarian cancer (39), knockdown of SR-B1 led to decreased GPx4 expression accompanied by increased PL oxidation and cell death (Fig. S3). To further study the importance of SR-B1-mediated transport of α-toc into cells and its impact on PL oxidation and ferroptosis, we used BLT-1. Interestingly, the addition of BLT-1 to Ramos, SUDHL4, and 786-O cells resulted in rescue of ML210-induced cell death in the absence of lipoprotein addition (Fig. S4), which suggests that ML210 uptake by these cancer cells involves SR-B1. The SR-B1 receptor is known to be involved in cellular drug delivery (40). Therefore, to determine SR-B1’s role in the uptake of α-toc, we used erastin, which directly targets cell surface x_c_^−^ (41) to induce cell membrane PL oxidation and ferroptosis. As demonstrated previously, treatment of Ramos, SUDHL4 and 786-O cells with α-toc HDL, α-toc LDL and α-toc ocHDL NPs rescued the cells from erastin-induced cell death (Fig. 2); however, blocking delivery of α-toc using BLT-1 resulted in cell death (Fig. 3*A*).

**Figure 3:**
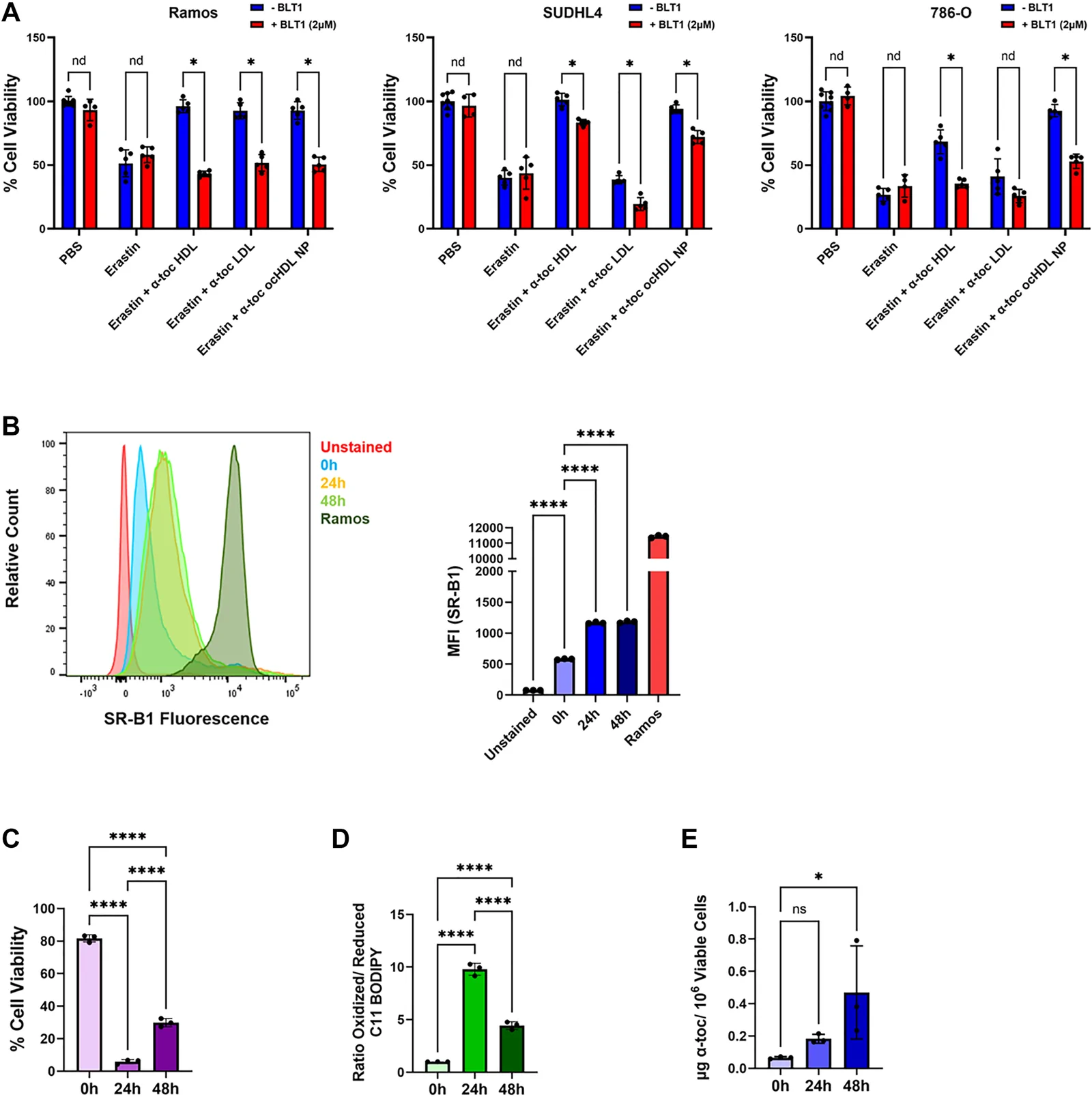
**SR-B1 mediates α-Toc rescue in cancer cells.***A*, rescue of Ramos, SUDHL4 and 786-O cells treated with erastin (Ramos–4 μM; SUDHL4–1 μM; 786-O–4 μM) by α-toc HDL (50 nM), α-toc LDL (50 nM) and α-toc ocHDL NPs (50 nM) in the presence or absence of BLT-1 (2 μM). *B*, SR-B1 expression by flow cytometry in U266B1 cells treated with ML210 (2 μM) for 0, 24 or 48 h. *C*, cell viability of U266B1 cells treated with ML210 (2 μM) for 0, 24 or 48 h. *D*, C11 BODIPY analysis of lipid peroxide accumulation in U266B1 cells treated with ML210 (2 μM). *E*, cellular α-toc levels in U266B1 cells treated with ML210 (2 μM) for 0, 24 or 48 h. Statistics: A. Student’s *t* test comparing -BLT1 and +BLT1. *B–E*. 1-way ANOVA with Tukey *post hoc* analysis. ∗*p* < 0.05 and ∗∗∗∗*p* < 0.0001.

To further investigate the role of SR-B1 in mediating the transport of α-toc into cancer cells, we identified the ferroptosis-sensitive, cholesterol auxotrophic multiple myeloma cell line U266B1 (42) as having negligible SR-B1 expression (Fig. 3*B*). Treatment of U266B1 cells with ML210 increased expression of SR-B1, and we measured an increase in lipid peroxide accumulation and cell death at 24 h of treatment (Fig. 3, *C* and *D*). Interestingly, lipid peroxide levels decreased, and cell viability increased at 48 h of treatment (Fig. 3, *C* and *D*), suggesting that increased SR-B1 expression in U266B1 cells exposed to ML210 enabled increased uptake of α-toc from serum lipoproteins. Indeed, α-toc levels in U266B1 cells rose at 48 h of ML210 treatment (Fig. 3*E*), corresponding to the decrease in lipid peroxide levels and increased cell viability.

Finally, we generated a 786-O cell line overexpressing SR-B1 (SR-B1 O/E). Over-expression was confirmed by Western blot and flow cytometry analyses (Fig. S5) in comparison to 786-O cells transfected with an empty plasmid (WT, with reference to SR-B1 expression). SR-B1 O/E cells had significantly more α-toc compared to WT cells at baseline (Fig. S5), which correlated to reduced sensitivity and higher IC50 values for ferroptosis inducers (ML210, RSL3 and Au-HDL NPs) in the SR-B1 O/E cells compared to WT controls (Fig. S5). Taken together, these results strongly implicate SR-B1 as a critical receptor for α-toc delivery to cancer cells to prevent ferroptosis.

To investigate the importance of LDLR-mediated transport of α-toc into cells, we employed siRNA to knockdown LDLR expression in 786-O cells, which was confirmed by flow cytometry (Fig. 4*A*). Reduction of LDLR did not significantly change SR-B1 (Figs. 4*A* and S3) or GPx4 expression (Fig. S3). Using the LDLR knockdown cells, we investigated whether α-toc containing HDL and LDL could rescue ML210-induced ferroptosis. The HDLs demonstrated rescue of cell death at the highest concentration (100 nM) in all groups, and the α-toc HDL particles demonstrated significant rescue of cell death at all concentrations in all groups (Fig. 4, *B*–*D*). For LDL, we found significant rescue in the siSCR and siLDLR groups, and the α-toc LDL demonstrated rescue in all groups (Fig. 4, *B*–*D*), most impressively at all α-toc LDL concentrations with siLDLR knockdown (Fig. 4*D*). Treatment with BLT-1 reduced the ability of α-toc LDL to rescue erastin-induced 786-O cell death (Fig. 4*E*), confirming that the enhanced rescue in the LDLR knockdown cells is due to promiscuity of lipoprotein binding and uptake of α-toc through SR-B1.

**Figure 4:**
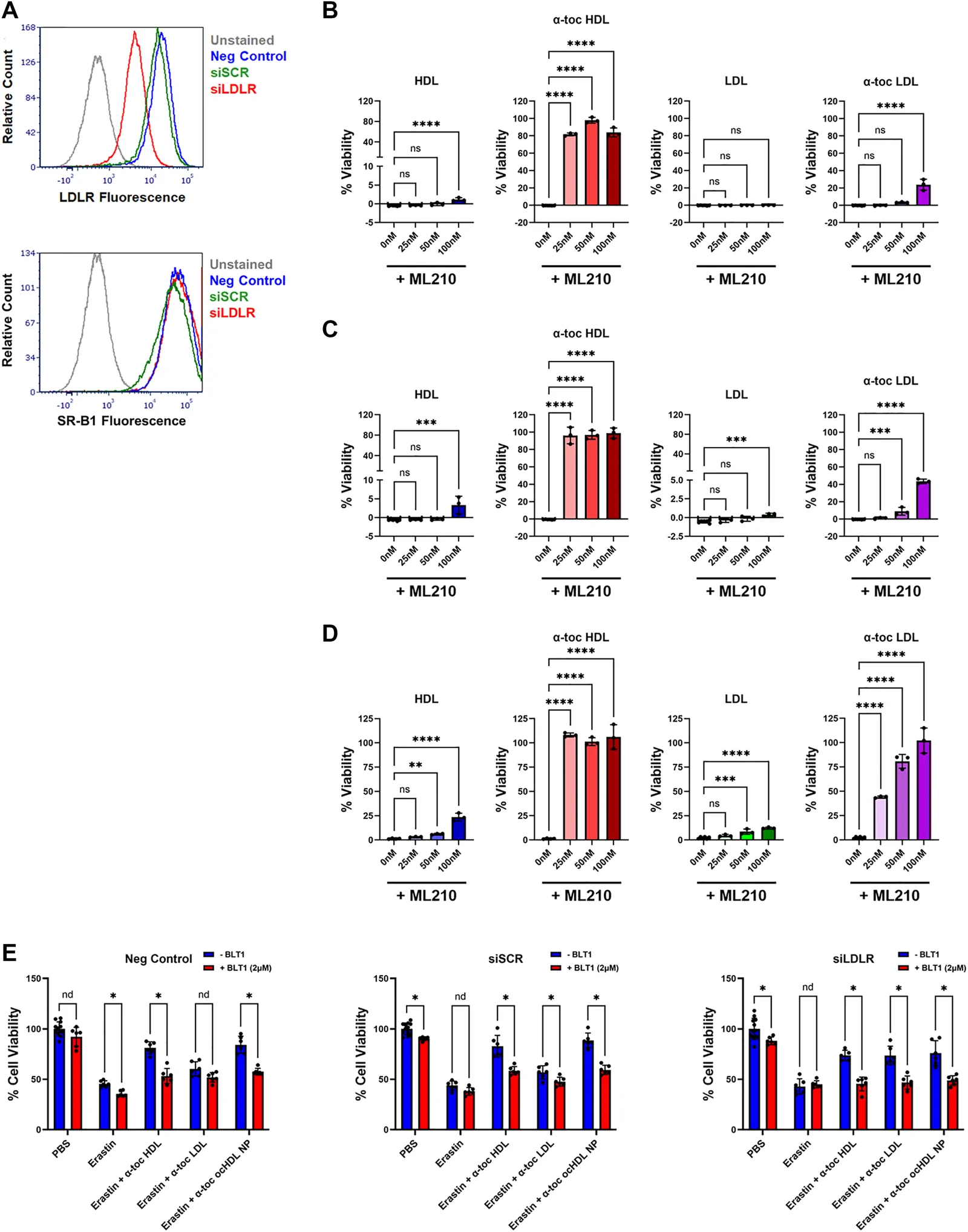
**LDLR does not mediate α-Toc rescue in cancer cells.***A*, flow cytometry analysis of LDLR (*top*) and SR-B1 (*bottom*) expression in 786-O cells treated with vehicle control (Neg Control), scrambled siRNA (siSCR) or siRNA against LDLR (siLDLR). *B–D*, rescue of ML210-induced (1 μM) cell death in 786-O neg control (*C*), siSCR (*D*) and siLDLR (*E*) cells with increasing concentrations of HDL, α-toc HDL, LDL, and α-toc LDL. *E*, rescue of 786-O Neg Control, siSCR and siLDLR cells treated with erastin (4 μM) by α-toc HDL (50 nM), α-toc LDL (50 nM) and α-toc ocHDL NPs (50 nM) in the presence or absence of BLT-1 (2 μM). Statistics: *B–D*. 1-way ANOVA with Tukey *post hoc* analysis. E. Student’s *t* test comparing -BLT1 and +BLT1. ∗*p* < 0.05, ∗∗*p* < 0.01, ∗∗∗*p* < 0.001 and ∗∗∗∗*p* < 0.0001 indicate statistical significance. No statistical difference (*p* > 0.05) is noted by “ns.”

To quantify cellular uptake of α-toc from lipoproteins, we employed a fluorescently labeled a α-toc molecule (BODIPY-α-toc). HDL, LDL, and ocHDL NPs were loaded with BODIPY-α-toc using the same protocol used for the synthesis of α-toc HDL, α-toc LDL and α-toc ocHDL NPs. Incorporation of BODIPY-α-toc was confirmed by fluorescence, with BODIPY-α-toc LDL and BODIPY-α-toc ocHDL NPs having 14.8 and 4.0 times the fluorescent signal (AU) per apolipoprotein relative to BODIPY-α-toc HDL (1.0; Table S2). Ramos, SUDHL4 and 786-O cells were treated with equal amounts of HDL, LDL and ocHDL NPs based on BODIPY-α-toc loading and analyzed by flow cytometry. Cellular delivery of BODIPY-α-toc was significantly higher by HDL and ocHDL NPs compared with LDLs, even though LDLs had significantly more BODIPY-α-toc per particle (Fig. S6). Addition of BLT-1 reduced uptake of BODIPY-α-toc delivered by HDL and ocHDL NPs but was less effective at reducing delivery by LDL, supporting that BODIPY-α-toc uptake is largely mediated by SR-B1 (Fig. S6). Taken together, these data indicate that SR-B1 is the critical receptor for α-toc delivery from lipoproteins and that delivery of α-toc *via* SR-B1 is responsible for rescue from cell membrane PL oxidation and ferroptosis.

### α-toc delivery by HDLs *via* SR-B1 rescues differentiated neuronal cells from ferroptosis

Lipid oxidative stress and ferroptosis is not limited to cancer cells. Control over ferroptosis by α-toc and GPx4 has been documented in other systems, such as the hematopoietic (16) and neurologic systems (15). Neuronal cell death by ferroptosis can result in neurodegenerative disease (43, 44). Further, SR-B1 and LDLR have both been implicated in central nervous system physiology and pathology, including neurodegeneration (45, 46). We therefore utilized an *in vitro* cell culture model of neurons to study the α-toc/HDL/SR-B1 axis. The neuroblastoma SH-SY5Y cell line can be differentiated *in vitro* by adding retinoic acid and brain derived neurotrophic factor (BDNF) with ultimate removal of serum in the cell culture media (see methods) to form non-proliferative neuron-like cells. The differentiated SH-SY5Y cells are a useful *in vitro* model to study neurodegenerative disorders. Differentiated SH-SY5Y cells experience ferroptosis-like cell death, including increased reactive oxygen species, lipid peroxidation, and cell death in the presence of 1-methyl-4-phenylpyridinium (MPP), which has been used as an *in vitro* model of Parkinson’s disease (47). Accordingly, we differentiated SH-SY5Y cells into neuronal-like cells as previously described (47). Notably, undifferentiated SH-SY5Y cells expressed SR-B1, while differentiated SH-SY5Y cells expressed little detectable SR-B1 (Fig. 5*A*). This is expected, as undifferentiated SH-SY5Y cells are a highly proliferative neuroblastoma cell line while terminally differentiated SH-SY5Y display a phenotype closer to neurons, where SR-B1 expression is reduced (48). Treatment of differentiated SH-SY5Y cells with MPP increased SR-B1 expression in a time dependent manner (Fig. 5*A*), and significantly increased PL oxidation (Fig. 5*B*). Addition of HDL with α-toc reduced lipid oxidation to baseline (Fig. 5*B*) and rescued the cells from cell death (Fig. 5*C*). Because the differentiated SH-SY5Y cells are grown in serum that does not contain lipoproteins, we attempted to add free α-toc to rescue the cells from lipid oxidation and cell death. However, free α-toc failed to reduce lipid oxidation and did not rescue the cells from MPP-induced cell death (Fig. 5, *B* and *C*). Presumably, this is due to the lack of lipoprotein carriers (HDL and LDL) that enable delivery of α-toc to the neurons *via* SR-B1. Taken together, these data demonstrate that differentiated SH-SY5Y cells in conditions of increased oxidative stress, increased SR-B1 expression, thereby enabling α-toc delivery *via* HDL and rescue of the neuronal cells.

**Figure 5:**
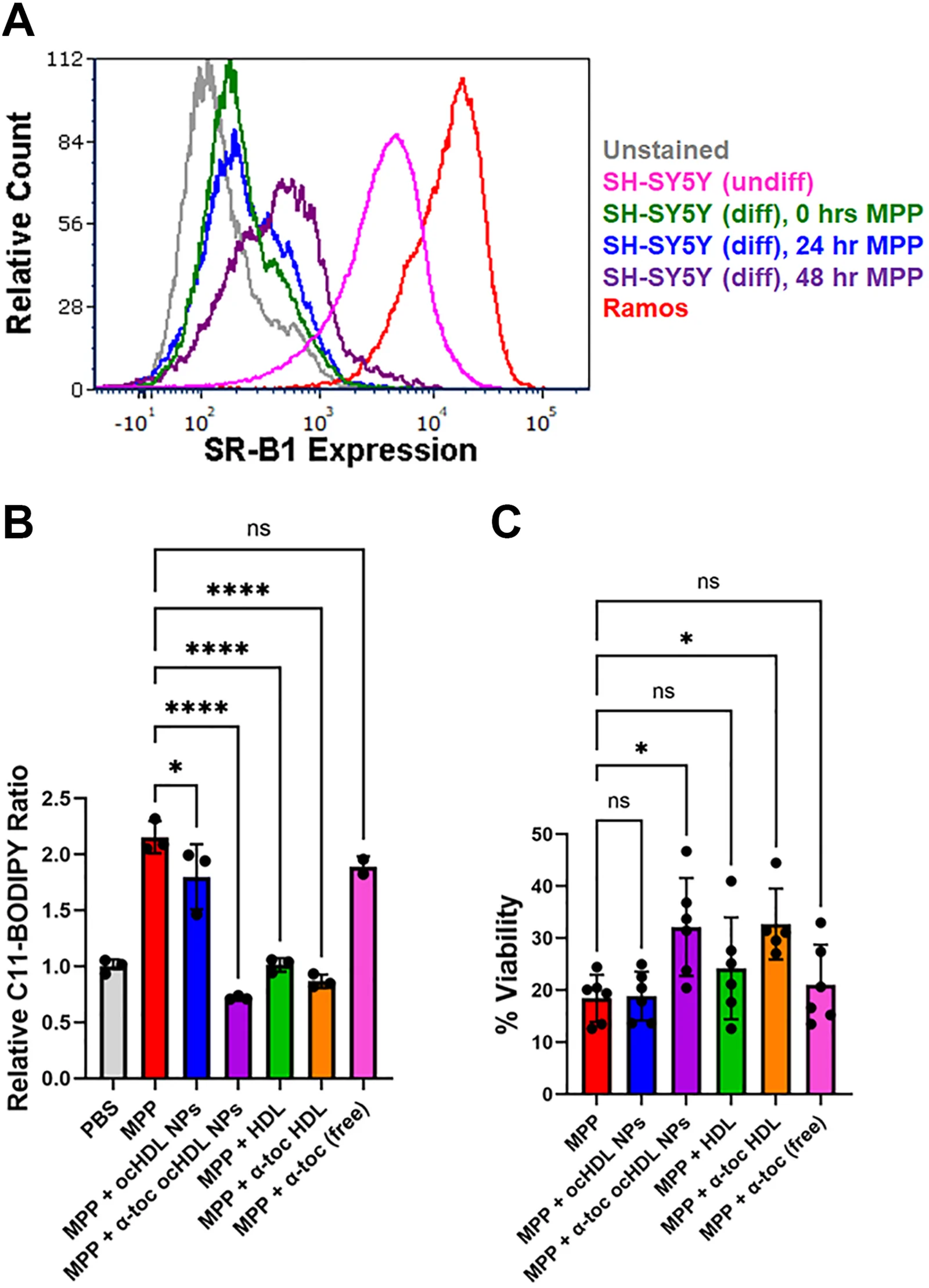
**MPP-induced ferroptosis-like cell death rescued in differentiated SH-SY5Y neuronal cells by α-toc HDLs.***A*, SR-B1 expression analysis by flow cytometry. Differentiated SH-SY5Y cells were treated with MPP (2.5 mM) for 0, 24, or 48 h prior to flow analysis. Ramos was used as an SR-B1 positive control. SH-SY5Y (undiff) = undifferentiated SH-SY5Y cells. SH-SY5Y (diff) = differentiated SH-SY5Y cells. *B*, SH-SY5Y cells were treated MPP (2.5 mM) in addition to 0 or 25 nM ocHDL NPs, α-toc ocHDL NPs, HDL, α-toc HDL or free α-toc (250 nM). PUFA-PL-OOH accumulation was quantified by C11 BODIPY flow cytometry 48 h later. Data presented as median fluorescent intensity (MFI). *C*, SH-SY5Y cells were treated with 2.5 mM MPP with 0 or 25 nM ocHDL NPs, α-toc ocHDL NPs, HDL, α-toc HDL or free α-toc (250 nM). Cell viability was quantified by MTS assay at 48 h. Statistics: *B* and *C*. 1-way ANOVA with Tukey *post hoc* analysis. ∗*p* < 0.05 and ∗∗∗∗*p* < 0.0001 indicate statistical significance. No statistical difference (*p* > 0.05) is noted by “ns.”

### Native and synthetic lipoproteins rescue cancer cells from synthetic gold nanoparticle-templated HDL NP (Au-HDL NPs) induced ferroptosis

We have previously demonstrated that Au-HDL NPs potently induce cell death in the SUDHL4 and Ramos cell lines (25), as well as other ferroptosis-sensitive cancers, such as ovarian cancer and acute myeloid leukemia [AML; (39, 49, 50)]. The Au-HDL NPs potently reduce GPx4 expression through depletion of intracellular selenium levels (49), leading to accumulation of phospholipid peroxides and ferroptotic cell death (25, 39). Additionally, cell death is rescued by adding a lipophilic antioxidant or iron chelator (25, 39, 49). Treatment of 786-O cells with Au-HDL NPs yielded similar results, including reduced GPx4 expression (Fig. S7), increased phospholipid peroxidation (Fig. S7), and ferroptotic cell death, which can be rescued by addition of the lipophilic antioxidant ferrostatin-1 and the iron chelator deferoxamine (DFO, Fig. S7). As observed with ML210 and erastin, we measured a dose-dependent rescue of Au-HDL NP-induced ferroptosis upon addition of HDL, α-toc HDL, α-toc LDL and α-toc ocHDL NP (Fig. S8). Rescue of cancer cell death correlated with decreased PL oxidation (Fig. S8). Importantly, rescue of cell death appeared independent of the reduction of GPx4 observed with the Au-HDL NP treatment (Fig. S9).

### Au-HDL NP treatment reduces cellular α-toc levels in cancer cells

Given the high affinity of Au-HDL NPs for SR-B1 (25, 39, 51, 52, 53), we investigated the effect of Au-HDL NP treatment on intracellular α-toc levels in Ramos, SUDHL4 and 786-O cells. Au-HDL NP treatment decreased cellular α-toc levels in Ramos, SUDHL4 and 786-O cells (Fig. 6, *A*–*C*). *In vivo* data show that delivery of α-toc to target cells rescues cells from ferroptosis independent of GPx4 expression (13, 14, 15, 16). Thus, we employed Au-HDL NPs, and their functional ability to reduce GPx4, but also incorporated α-toc into the synthesis of Au-HDL NPs (α-toc Au-HDL NPs) to mechanistically validate the *in vivo* findings using a single, targeted particle. The surface phospholipid layer of the Au-HDL NP is amenable to binding and loading hydrophobic molecules typically carried by HDLs (54). Synthesized α-toc Au-HDL NPs were similar in size and surface charge to Au-HDL NPs and contained 5.0 ± 1.0 molecules of α-toc/ApoA-I (Table S3). In contrast to Au-HDL NPs, α-toc Au-HDL NPs did not decrease cellular α-toc levels in Ramos, SUDHL4 and 786-O cells (Fig. 6, *A*–*C*). Functionally, the α-toc Au-HDL NPs significantly improved the viability of Ramos, SUDHL4 or 786-O cells compared with Au-HDL NPs (Fig. 6, *D*–*F*). Correspondingly, α-toc Au-HDL NP rescued the cell membrane of each of the cell lines from increased PL oxidation (Fig. S10). Importantly, GPx4 protein expression was reduced by both α-toc Au-HDL NP and Au-HDL NP, but cell viability rescue was restricted to those cells treated with the α-toc Au-HDL NP (Fig. S10). Finally, to verify that the α-toc delivery by Au-HDL NPs was *via* SR-B1, we co-treated Ramos, SUDHL4 and 786-O cells with a combination of Au-HDL NPs or α-toc Au-HDL NPs with BLT-1. The addition of BLT-1 prevented the rescue achieved by incorporation of α-toc into Au-HDL NPs (Fig. 6, *G*–*I*). Taken together, these results clearly demonstrate that Au-HDL NP increase PL oxidation and induces ferroptosis by a combined mechanism that includes reduced cellular α-toc and GPx4, while α-toc delivery to target cells *via* SR-B1 regulates cell sensitivity to ferroptosis.

**Figure 6:**
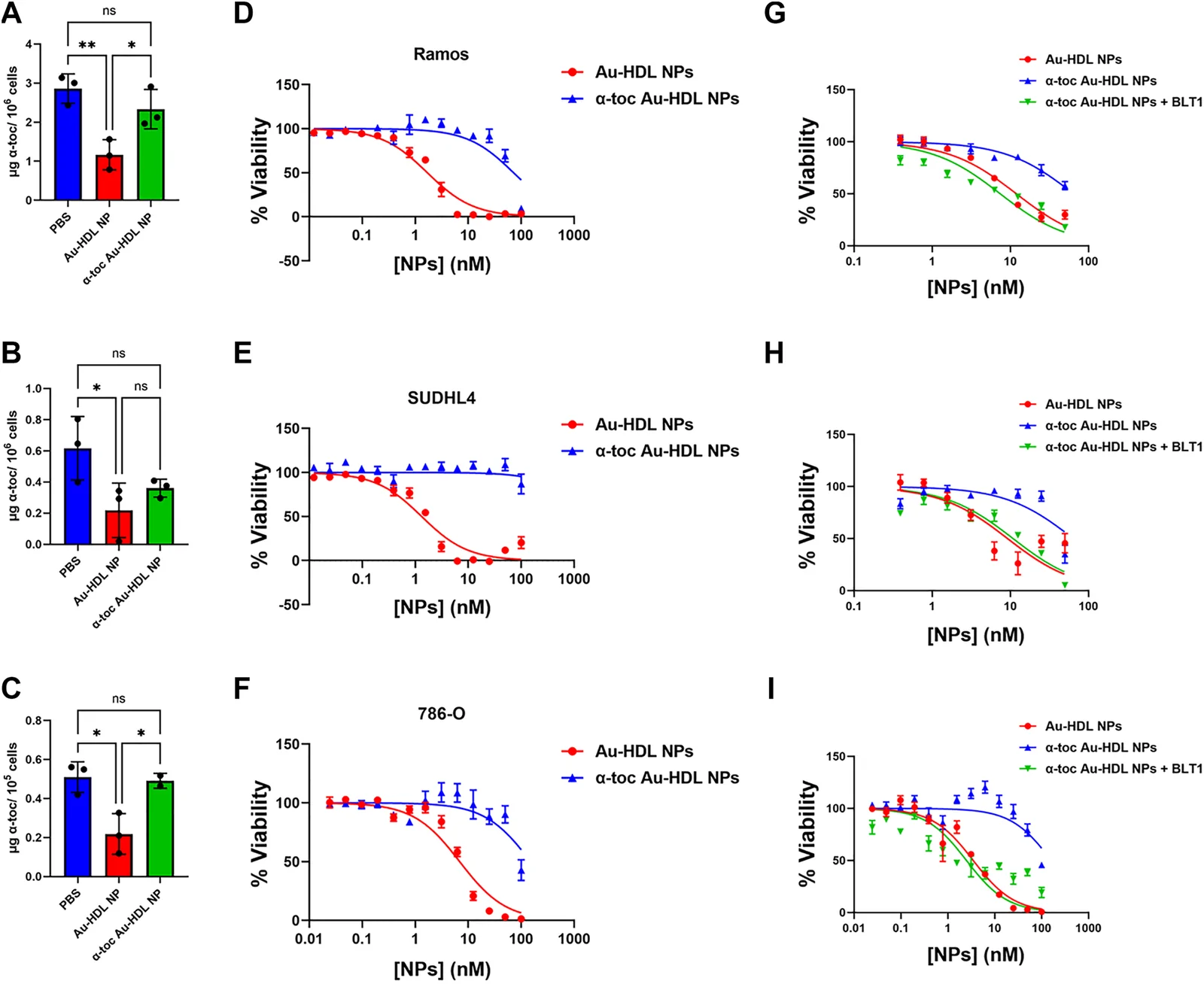
**Incorporation of α-toc into Au-HDL NPs rescues DLBCL and ccRCC cells from ferroptosis.***A–C*, cellular α-toc levels in Ramos (*A*), SUDHL4 (*B*) and 786-O (*C*) cells following 24 h treatment with Au-HDL NPs (10 nM) or α-toc Au-HDL NPs (10 nM). *D* and *F*, cell viability (MTS) assay for Ramos (*D*), SUDHL4 (*E*) and 786-O (*F*) cells treated with Au-HDL NPs or α-toc Au-HDL NPs for 72 or 120 h. *G–I*, cell viability (MTS) assay for Ramos (*G*), SUDHL4 (*H*) and 786-O (*I*) cells treated with Au-HDL NPs, α-toc Au-HDL NPs or α-toc Au-HDL NPs + BLT-1 (Ramos, SUDHL4–2 μM; 786-O–20 μM) for 72 or 120 h. Statistics: *A–C*. 1-way ANOVA with Tukey *post hoc* analysis. ∗*p* < 0.05 and ∗∗*p* < 0.01 indicate statistical significance. No statistical difference (*p* > 0.05) is noted by “ns.”

## Discussion

We report an α-toc/HDL/SR-B1 axis of cell membrane phospholipid redox control whereby lipoproteins, predominantly HDLs, carry α-toc, bind to SR-B1, and deliver α-toc to the cell membrane to modulate oxidative damage to cell membrane phospholipids and cell death by ferroptosis. Both natural HDL and synthetic HDL NP ligands that target and bind SR-B1 can be used to manipulate the α-toc/HDL/SR-B1 axis to control lipid oxidation and cell fate.

Most studies directed at identifying and understanding mechanisms of ferroptosis have been conducted using *in vitro* cell culture systems (9, 24, 55, 56, 57, 58). These discoveries have provided powerful insight into the cellular mechanisms of ferroptosis and identified targeted small molecules that inhibit certain pathways (*e.g.*, erastin targeting system x_c_^−^ and RSL3 or ML210 inhibiting GPx4) to induce ferroptosis. Additionally, cell death by ferroptosis is classically inhibited by chelating intracellular iron (*e.g.*, deferoxamine) and by adding cell exogenous lipophilic antioxidants (*e.g.*, α-toc or Liproxstatin-1). Notably, α-toc is a natural, physiologically relevant lipophilic RTA (59). These competing factors have resulted in an appreciation that multiple mechanisms of ferroptosis must be employed simultaneously to effectively actuate this mechanism of cell death (60). The Au-HDL NP is unique in its ability to induce ferroptosis *in vitro* and *in vivo* by effectively binding SR-B1, reducing cellular α-toc delivery, and reducing GPx4 expression.

Opportunities for manipulating or therapeutically targeting mechanisms of ferroptosis *in vivo* are limited. *In vivo*, essential vitamins (*e.g.*, α-toc) and trace elements regulate cell sensitivity to ferroptosis. For example, selenium is an essential micronutrient that critically regulates the synthesis of redox selenoproteins, like GPx4. Selenium delivery to cells is tightly regulated and is required for selenoprotein production and resistance to ferroptosis (61). Similarly, α-toc is an essential vitamin that provides resistance to ferroptosis. Following dietary absorption in the gut, α-toc requires solubilization and transport in the blood stream by lipoproteins (17, 18). Specifically, LDL (17, 62, 63) and HDL (17, 64, 65) are natural nanoparticles that carry α-toc in the circulation for delivery to target cells. The LDL particles target the LDL receptor (LDLR) whereupon the LDL/LDL-R complex is internalized in a clathrin-dependent manner (20, 66). The LDL particle is then destined for degradation in the endolysosome whereby degradation products are distributed to the cell (20). The receptor for HDLs, SR-B1, was initially discovered as a high-affinity receptor for acetylated LDL; however, it was later discovered to be a preferential receptor for HDL (34). Lipid-rich HDLs bind SR-B1 and oligomerize SR-B1 at the cell membrane (67), where upon binding, HDLs deliver and exchange lipophilic cargo at the cell membrane (67, 68). Our data suggest that the direct exchange of lipophilic cargo enabled by SR-B1 after binding lipoproteins rich in α-toc is a critical mechanism for delivering α-toc to the plasma membrane where it can act to reduce PL oxidation and prevent ferroptosis.

In cancer, as suggested by our work (25, 39, 49, 50) and the work of others (56), targeting multiple mechanisms through which cancer cells protect themselves from oxidative damage and ferroptosis is essential to realizing successful therapy. Recent work in AML demonstrated that a FLT3 (FMS-like tyrosine kinase 3) inhibitor, gilteritinib, inhibits selenoprotein translation leading to ferroptosis (69). Interestingly, the authors found that gilteritinib efficacy was significantly reduced by dietary vitamin E (69), further highlighting the complementary nature of selenoproteins, such as GPx4, and α-toc in protecting cells from ferroptosis. We demonstrate here that targeting SR-B1 with a high-affinity synthetic HDL devoid of α-toc (Au-HDL NPs) leads to a reduction in cellular α-toc levels that, along with simultaneous reduction of GPx4, induces ferroptosis. Ultimately, given the significant effect that serum α-toc has in ameliorating small molecule drug-induced ferroptosis, the α-toc/HDL/SR-B1 axis is of particular importance as a therapeutic target. In neurodegenerative diseases, the goal is the opposite, since ferroptosis is thought to be a major mediator of neurodegeneration and inhibition of ferroptosis is the goal.

Overall, the α-toc/HDL/SR-B1 axis of cell membrane PL redox control that we describe herein may enhance our understanding of HDL function and biology, cell membrane redox balance, and ferroptosis to provide next generation therapies for cancer, neurodegeneration, inflammation, and other diseases associated with increased lipid oxidative damage.

## Experimental procedures

### Cell lines and reagents

The Ramos, SUDHL4, 786-O, U266B1 and SH-SY5Y cell lines were purchased directly from ATCC and used within 3 months of resuscitation from cryopreservation. ATCC uses short tandem repeats (STR) profiling to authenticate their cell lines. B-cell lymphoma and ccRCC cell lines were cultured in RMPI 1640 media (Corning), supplemented with 10% fetal bovine serum (FBS, Corning) and 1% PenStrep (Corning). ML210 and erastin were obtained from Selleck Chemicals. BLT-1 and MPP+ (1-methyl-4-phenylpyridinium iodide) were obtained from Sigma-Aldrich. The C11-BODIPY dye was obtained from ThermoFisher Scientific. HDL and LDL were obtained from Sigma-Aldrich. Molar concentrations of HDL were calculated as described previously (53). Molar concentrations of LDL were calculated by converting the mg/ml to molarity, assuming that the entire protein concentration is comprised of apoB-100 and that there is a single apoB-100 protein per LDL.

### Loading of HDL and LDL with α-toc and BODIPY-α-toc

HDL and LDL were incubated with free α-toc (dissolved in DMSO) at a molar ratio of 10/1 α-toc/apoA-I (HDL) or apoB-100 (LDL), for 4 h at room temperature on an orbital shaker (60 rpm). Following incubation, excess, unincorporated α-toc was removed by centrifugation using spin filter columns (50 kDa MWCO). Protein concentrations were determined using the BCA assay (Pierce BCA protein assay kit, ThermoFisher Scientific).

Similarly, BODIPY-α-toc was dissolved in DMSO and added to HDL and LDL using the same molar ratios as above. Following 4 h incubation at room temperature, the BODIPY-α-toc HDL and LDL were purified by centrifugation using spin filter columns.

### Synthesis of HDL NPs

ocHDL NPs and α-toc ocHDL NPs were synthesized using a modified in-house protocol (21). Briefly, apolipoprotein A-I (apoA-I; MyBioSource) and sodium cholate (final concentration 19 mM; Sigma-Aldrich) in water were combined with the organic core scaffold (3-(stearoyloxy)-2,2-bis[(stearoyloxy)methyl]propyl stearate; PE-S4; CAS 115-83-3; Sigma-Aldrich), phospholipids (1,2-dioleoyl-sn-glycero-3-phosphocholine; DOPC; Avanti Polar Lipids) and α-toc (Sigma-Aldrich) in dichloromethane (DCM). Reagents were added at a ratio of 300/5/2/1 DOPC/α-toc/apoA-I/PE-S4, with the final resultant mixture 75% water/25% DCM. An emulsion was generated by successive rounds of vortexing and sonication, followed by stirring to allow the organic phase (DCM) to evaporate and the ocHDL NPs to self-assemble. The resultant nanoparticle solution was then dialyzed against 1 X PBS for 24 h, with one buffer change at ∼12 h. ApoA-I concentration was calculated using a BCA assay, and the final nanoparticle concentration determined using 2.23 apoA-I/nanoparticle. BODIPY-α-toc ocHDL NPs were synthesized using the same protocol as α-toc ocHDL NPs.

Au-HDL NPs were synthesized using standard protocols, as described previously (25, 39). Briefly, 5 nm gold nanoparticles (AuNP, NanoComposix) were surface-functionalized with apoA-I and the phospholipids DPPC (1,2-dipalmitoyl-sn-glycero-3-phosphocholine, Avanti Polar Lipids) and PDP PE (1,2-dipalmitoyl-sn-glycero-3-phosphoethanolamine-N-[3-(2-pyridyldithio)propionate], Avanti Polar Lipids) at a molar ratio of 250/250/5/1 for DPPC/PDP PE/apoA-I/AuNP. Au-HDL NPs were purified by tangential flow filtration (TFF), and the concentration determined using UV/Vis spectrometry and Beer’s law. α-toc was incorporated into Au-HDL NPs at a 20/1 α-toc/AuNP ratio during synthesis.

### Characterization of lipoproteins and HDL NPs

All lipoproteins (*e.g.*, HDL, LDL), ocHDL NPs and Au-HDL NPs were characterized by dynamic light scattering (DLS) to measure size (diameter, in nm), surface charge (zeta potential, reported in mV) and α-toc content (Vitamin E assay kit, MyBioSource, MBS2540415). DLS (3 runs per construct, with each run consisting of 12 reads of 15 s each; sample concentration of ∼40 nM in 1 X PBS) and zeta potential (3 runs per construct, with each run consisting of a minimum of 30 to a maximum of 100 measurements; sample concentration of ∼40 nM in water) were measured using a Malvern Zetasizer. A UV/Vis spectrophotometer was used to quantify the absorbance readings (533 nm) from the Vitamin E Assay Kit (N = 3 per lipoprotein/nanoparticle).

The number of BODIPY-α-toc per apolipoprotein was quantified using a fluorescent plate reader. As BODIPY fluorescence changes based on its environment (*e.g.*, highly fluorescent in a lipid membrane versus low/nonexistent fluorescence in an aqueous buffer), the loading of BODIPY-α-toc is reported relative to the quantity present in BODIPY-α-toc HDL.

### Quantification of lipoprotein receptors by flow cytometry

SR-B1 and LDLR expression was quantified by flow cytometry, as described previously (25). APC-labeled SR-B1 antibody was obtained from BioLegend (#363208) and APC-labeled LDLR antibody was obtained from Abcam (AB275614). Blocking was achieved using Human TruStain FcX (BioLegend).

### Quantification of PUFA-PL-OOH accumulation

Measurement of PUFA-PL-OOH accumulation was conducted using the C11-BODIPY fluorescent dye and flow cytometry, as described previously (25, 39). C11-BODIPY was obtained from ThermoFisher Scientific (D3861).

### Western blot analysis

Western blots were conducted as described previously (25, 39). Antibodies against SR-B1 (ab52629, Abcam) and GPx4 (ab125066, Abcam) were used at a 1:1000 dilution; the antibody against β-actin (#4967, Cell Signaling Technologies) was used at a 1:3000 dilution; and the secondary antibodies (BioRad) were used at a 1:2000 dilution. Blots were imaged using an Azure 300 Western blot imager (Azure Biosystems).

### Cell viability assay (MTS)

Cell viability was quantified using the MTS assay, as described previously (25, 39). Briefly, Ramos and SUDHL4 cells were plated at a density of 2 × 10^5^ cells/ml for experiments lasting 72 h (cell death induced by ML210, Au-HDL NPs) and 1 × 10^6^ cells/ml for experiments lasting 24 h (cell death induced by erastin). 786-O cells were plated at a density of 5 × 10^3^ cells/ml for 72 h experiments (ML210, Au-HDL NPs), and 1 × 10^4^ cells/ml for 24 h experiments (erastin). Promega’s CellTiter Aqueous reagent (Promega) was used to quantify percent viability, as described previously (25, 39).

### Knockdown of SR-B1 and LDLR in 786-O cells

786-O cells were transfected with vehicle control (neg control), 50 nM scrambled siRNA (siSCR; Invitrogen), 50 nM SR-B1 siRNA (siSR-B1; Invitrogen) or 50 nM LDLR siRNA (siLDLR; Invitrogen) using Lipofectamine RNAiMAX (Invitrogen), as described previously (70). Cells were cultured for 48 h prior to replating and treated with various ferroptosis inducers (ML210) and lipoproteins. Knockdown of LDLR was confirmed by flow cytometry.

### Generation of SR-B1 overexpressing 786-O cells

For SR-B1 overexpression of 786-O cells, pLenti6.3/V5-DEST-SCARB1 [DNASU Plasmid Clone HsCD00942131; a gift from the Center for Personalized Diagnostics; (71)] was transfected into HEK293 t cells along with pMD2.G envelope and psPAX2 packaging vectors (gifts from Didier Trono (Addgene plasmids #12259 and #12260) using PEI to generate lentivirus. Alongside, pLenti6.3/V5-DEST-GFP [Addgene Plasmid # 40125; a gift from Lynda Chin; (72)] was used to generate control cell lines. Medium was changed the following day, and lentivirus-containing media was collected 48 h post-transfection and filtered using a 0.45 μm sterile filter. 786-O cells were infected using lentivirus-containing media supplemented with 8 μg/ml polybrene, and the media was changed ∼18 h later. After 48 h, 5 μg/ml blasticidin (InvivoGen an-bl-1) was used to select for transduced cells.

### Differentiation of SH-SY5Y cells

SH-SY5Y cells were differentiated as described previously (47). Briefly, SH-SY5Y cells were cultured with 10 μM retinoic acid in DMEM/Ham’s media (Corning) supplemented with 10% FBS, 2 mM glutamine and 1% PenStrep for 7 days, followed by culture in serum free DMEM/Ham’s media with 2 mM glutamine, 1% PenStrep and 50 ng/ml BDNF (R&D Systems) for a further 5 to 6 days. After differentiation, SH-SY5Y cells were plated (4 × 10^5^ cells/ml) into collagen I coated six well plates (C11 BODIPY and SR-B1 flow analysis) or 96 well plates (MTS assay). Cells were allowed to adhere overnight prior to treatment with MPP+ (2.5 mM) and lipoproteins. Cells were cultured for 48 h prior to assaying for PUFA-PL-OOH accumulation, SR-B1 expression and viability.

### Cellular α-toc quantification

Ramos, SUDHL4 and 786-O cells were treated with Au-HDL NPs (10 nM), α-toc Au-HDL NPs (10 nM) or PBS for 24 h prior to harvesting. The viable cell number was quantified using the Countess cell counter (Invitrogen). Cellular α-toc was extracted using a chloroform/water mixture and sonication. α-toc content of the extracts was measured using the Vitamin E assay kit (MyBioSource), as described earlier.

### Statistical analysis

Statistics were run in GraphPad Prism 10. N values varied between 4 to 10 for cell viability/MTS assays (exact numbers indicated in each caption), while N = 3 for C11 BODIPY experiments. T-tests and 1-way ANOVAs, with multiple comparisons, were used for these studies. For MTS studies measuring the ability of lipoproteins to rescue small molecule–induced cell death (*e.g.*, HDL rescue of ML210-induced cell death) as well as the corresponding C11-BODIPY analysis of lipid peroxide accumulation, 1-way ANOVAs with multiple comparisons to the small molecule treatment alone were conducted. For MTS studies measuring the ability of BLT-1 to inhibit lipoprotein rescue of erastin- or ML210-induced cell death, multiple unpaired t-tests were run comparing each treatment condition without BLT1 (-BLT1) to that with BLT1 (+BLT1).

## Data availability

The data generated in this study are available on request from the corresponding authors.

## Supporting information

This article contains supporting information.

## Conflict of interest

C. S. T. and L. I. G. are cofounders of a biotechnology company that licensed the HDL NP technology from Northwestern University. The remaining authors, J. S. R., A. E. C., S. M. L., A. M., S. T. N. and A. Y. L., declare no competing interests. The remaining authors declare that they have no conflicts of interest with the contents of this article.
