# Supplementary material for: Regulation of lipid oxidation and ferroptosis by a tunable axis of vitamin E, high-density lipoprotein, and scavenger receptor class B type 1
Source: J Biol Chem. 2026 Aug 27;302(10):113497. doi: 10.1016/j.jbc.2026.113497 (PMC13631611; doi:10.1016/j.jbc.2026.113497)
Supplement: Supplementary file 1 [file mmc1.docx]

**SUPPORTING INFORMATION**

Regulation of Lipid Oxidation and Ferroptosis By a Tunable Axis of Vitamin E, High-Density Lipoprotein, and Scavenger Receptor Class B Type 1

Jonathan S. Rink, Andrea E. Calvert, Sophia M. Lamperis, Alexandra Moxley, Adam Yuh Lin, SonBinh T. Nguyen, Leo I. Gordon^*^, and C. Shad Thaxton^*^

J.S.R., A.E.C., S.M.L., A.M., C.S.T. Department of Urology, Northwestern University Feinberg School of Medicine, Chicago, IL, 60611, USA.

J.S.R., A.Y.L., L.I.G. Department of Medicine, Division of Hematology/ Oncology, Northwestern University Feinberg School of Medicine, Chicago, IL, 60611, USA.

J.S.R., A.Y.L., L.I.G., C.S.T. Robert H. Lurie Comprehensive Cancer Center, Northwestern University, Chicago, IL, 60611, USA.

S.T.N. Department of Chemistry, Northwestern University, Evanston, IL, 60208, USA.

C.S.T. Jesse Brown Veteran Affairs Medical Center, Chicago, IL, 60612, USA.

*Leo I. Gordon, MD	*C. Shad Thaxton, MD, PhD

Division of Hematology/Oncology	Department of Urology

541 N Fairbanks Court	303 E Superior St

Suite 2300	Lurie Building, 10-113

Chicago, IL 60611	Chicago, IL 60611

[l-gordon@northwestern.edu](mailto:l-gordon@northwestern.edu)	[cthaxton003@northwestern.edu](mailto:cthaxton003@northwestern.edu)

**Figure S1: SR-B1 and LDLR Expression.** Flow cytometry analysis of SR-B1 (A) and LDLR (B) expression in the lymphoma cell lines Ramos and SUDHL4 and the clear cell renal cell carcinoma (ccRCC) cell line, 786-O.

**Table S1: Characterization of HDL and LDL Nanoparticles**

**^‡^**Statistics: N = 3 per group; 1-way ANOVA, F = 3.201, degrees of freedom = 2, p=0.1132, R^2^ = 0.5162. ocHDL NPs vs. HDL p=0.1817. α-toc ocHDL NPs vs. HDL p=0.1299. **^†^**Statistics: N = 3 per group; Unpaired t test, two tailed. ocHDL NPs vs. α-toc ocHDL NPs p=0.0152.

**Figure S2: ML210 Induces Cell Death in DLBCL and ccRCC Cell Lines.** A-C. ML210 kill curves for Ramos (A), SUDHL4 (B) and 786-O (C) cells. Red arrow indicates concentration used in the α-toc lipoprotein rescue experiments detailed in Figures 1 and 2.

**Figure S3: Knockdown of SR-B1 Induces Ferroptosis in 786-O Cells.** A, B. siRNA mediated knockdown of SR-B1 (siSR-B1) reduced both SR-B1 (A) and GPx4 (B) expression in 786-O cells. C. PUFA-PL-OOH accumulation increases with knockdown of SR-B1 in 786-O cells. ****p<0.0001. D. Knockdown of SR-B1 by siRNA reduced 786-O cell viability at 5 days post knockdown. ****p<0.0001.

**Figure S4: BLT-1 Treatment Rescues Cells from ML210-Induced Cell Death.** Ramos, SUDHL4 and 786-O cells were treated with PBS or ML210 (250 nM for Ramos; 100 nM for SUDHL4; 1 µM for 786-O) in the presence or absence of BLT-1 [(-) BLT-1, 1 µM]. Viability was quantified by MTS assay. Data are presented relative to PBS control (-) BLT-1. Statistics: N = 5 per group. Multiple unpaired t-tests [(-) BLT-1 vs (+) BLT-1; 2 tests performed per cell line]. Ramos p values: PBS = 0.987064, ML210 < 0.000001. SUDHL4 p values: PBS = 0.000097, ML210 = 0.000032. 786-O p values: PBS = 0.013589, ML210 < 0.000001.

**Figure S5: Over-expression of SR-B1 in 786-O Cells Increases Cellular α-toc Levels and Reduces Sensitivity to Ferroptosis Inducers.** A. Western blot (left) and flow cytometry (right) analysis of SR-B1 expression in 786-O wild type (WT) and SR-B1 over-expressing (SR-B1 O/E) cells. GPx4 expression was unchanged with SR-B1 over-expression. B. Cellular α-toc levels were increased in SR-B1 O/E cells compared to WT. C. SR-B1 O/E cells were more resistant to ferroptosis inducers ML210 (top), RSL3 (middle) and Au-HDL NPs (bottom) compared to WT cells. IC50s: ML210 – WT = 83.47 nM, SR-B1 O/E = 295.4 nM; RSL3 – WT = 0.2455 nM, SR-B1 O/E = 7.417 nM; Au-HDL NP – WT = 1.972 nM, SR-B1 O/E = 47.93 nM.

**Table S2: Characterization of BODIPY-α-toc HDL and LDL Nanoparticles**

**Figure S6: Uptake of BODIPY-α-toc via Lipoproteins.** A-C. Median fluorescent intensities (MFI) of BODIPY in Ramos (A), SUDHL4 (B) and 786-O (C) cells treated with PBS, BODIPY-α-toc HDL, BODIPY-α-toc LDL or BODIPY-α-toc ocHDL NPs for 3 hrs. **p<0.01. ***p<0.001. ****p<0.0001. D-F. MFI of BODIPY in Ramos (D), SUDHL4 (E) and 786-O (F) cells treated with BODIPY-α-toc loaded lipoproteins with (red) and without (blue) the addition of 2 µM BLT-1 for 3 hrs. **p<0.01. ****p<0.0001.

**Figure S7:** **Au-HDL NPs Induce Ferroptosis in Clear Cell Renal Cell Carcinoma Cells.** A. Western blot of 786-O cells treated with increasing concentrations of Au-HDL NPs for 72 hrs. Actin was used as a loading control. B. Representative histogram of 786-O cells treated with Au-HDL NPs (10 nM) for 72 hrs prior to C11-BODIPY staining. C. Cell viability assay (MTS) of 786-O cells treated with increasing concentrations of Au-HDL NPs alone or in combination with Ferrostatin-1 (1 µM) or DFO (1 µM). *p<0.05 vs. Au-HDL NPs.

**Figure S8: α-toc Delivery by Lipoproteins Rescues Au-HDL NP-Induced Cell Death in DLBCL and ccRCC Cells.** A-C. Ramos (A), SUDHL4 (B) and 786-O (C) cells were treated with Au-HDL NPs (20nM) in combination with increasing concentrations of HDL, α-toc HDL, LDL, α-toc LDL, ocHDL NP and α-toc ocHDL NP for 72 hrs. *p<0.05. **p<0.01. ***p<0.001. ****p<0.0001. D. C11 BODIPY analysis of lipid peroxide accumulation in Ramos cells treated with Au-HDL NPs (10 nM) and HDL, α-toc HDL, LDL, α-toc LDL, ocHDL NP and α-toc ocHDL NP. *p<0.05. ****p<0.0001.

**Figure S9: hHDL and ocHDL NPs Cannot Rescue Au-HDL NP-Induced GPx4 Down-Regulation.** Ramos (A), SUDHL4 (B) and 786-O (C) cells were treated with Au-HDL NPs (10 nM) in combination with hHDL, α-toc hHDL, oc-HDL NPs or α-toc oc-HDL NPs at the listed concentrations for 48 hrs (A, B) or 72 hrs (C), followed by western blot analysis of GPx4 expression. Actin was used as a loading control.

**Table S3:** **Characterization of Au-HDL NPs**

**Figure S10: α-toc Au-HDL NPs Reduce GPx4 Expression But Do Not Induce Lipid Peroxide Accumulation.** A-C. PUFA-PL-OOH accumulation measured by C11 BODIPY. Ramos (A), SUDHL4 (B) and 786-O (C) cells were treated with Au-HDL NPs or α-toc Au-HDL NPs for 48 (Ramos, SUDHL4) or 72 (786-O) hrs prior to assessment of PUFA-PL-OOH accumulation by C11 BODIPY staining and flow cytometry. ****p<0.0001. D-F. Western blot analysis of GPx4 expression in Ramos (D), SUDHL4 (E) and 786-O (F) cells. GPX4 expression decreased with Au-HDL NP treatment, regardless of the presence of α-toc. Actin was used as a loading control.
